# The feasibility of decreasing the thresholds for biopsy in Kwak and C TIRADSs

**DOI:** 10.3389/fonc.2023.1027802

**Published:** 2023-02-08

**Authors:** Chao Fu, Yiyang Cui, Jing Li, Yan Wang, Caifeng Si, Kefei Cui

**Affiliations:** ^1^ Department of Ultrasound, The First Affiliated Hospital of Zhengzhou University, Zhengzhou, China; ^2^ Department of Interventional Radiology, The First Affiliated Hospital of Zhengzhou University, Zhengzhou, China

**Keywords:** thyroid nodules, ultrasound, cytology aspiration, sub-centimeter, thyroid imaging reporting and data system (TIRADS)

## Abstract

**Objectives:**

To estimate the feasibility of decreasing the original thresholds for biopsy in the Kwak Thyroid Imaging Reporting and Data System (Kwak TIRADS) and Chinese Thyroid Imaging Reporting and Data System (C TIRADS).

**Methods:**

This retrospective study included 3,201 thyroid nodules from 2,146 patients with a pathological diagnosis. We lowered the original fine-needle aspiration (FNA) thresholds with the TR4a-TR5 in Kwak and C TIRADSs and calculated the ratio of additional benign-to-malignant nodules being biopsied (RABM). If the RABM is less than 1, the decreased FNA thresholds could be accepted and used to the modified TIRADSs (modified C and Kwak TIRADSs). Then, we estimated and compared the diagnostic performance between the modified TIRADS and the original TIRADS to determine if the decreased thresholds could be an effective strategy.

**Results:**

A total of 1,474 (46.0%) thyroid nodules were diagnosed as malignant after thyroidectomy. The TR4c-TR5 in Kwak TIRADS and TR4b-TR5 in C TIRADS had a rational RABM (RABM < 1). The modified Kwak TIRADS had higher sensitivity, a positive predictive value, a negative predictive value, lower specificity, an unnecessary biopsy rate, and a missed malignancy rate compared with the original Kwak TIRADS (94.1% vs. 42.6%, 59.4% vs. 44.6%, 89.9% vs. 52.8%, 45.0% vs. 54.9%, 40.6% vs. 55.4%, and 10.1% vs. 47.1%, respectively, *P* < 0.05 for all). Similar trends were seen in the modified C TIRADS versus the original C TIRADS (95.1% vs. 38.7%, 61.7% vs. 47.8%, 92.3% vs. 55.0%, 49.7% vs. 64.0%, 38.3% vs. 52.2%, and 7.7% vs. 44.9%, respectively, *P* < 0.05 for all).

**Conclusions:**

The biopsy of all nodules with TR4C-TR5 in the Kwak TIRADS and TR4B-TR5 in the C TIRADS might be an effective strategy. This paper contributes to the contradiction concerning whether to perform FNA for the nodules smaller than 10 mm.

## Introduction

Thyroid ultrasonography is now regularly performed in clinical practice, and thyroid nodules are exceedingly common on ultrasound (US) with as many as 68% of adults having one (1, 2). Many international societies have reported that their own risk stratification systems (RSSs) used different US features and size thresholds to manage thyroid nodules (3–6). Currently, a 10 mm size threshold is widely used to determine whether to biopsy a thyroid nodule with suspicious features (3, 5, 7). In some researchers, however, it is supported that biopsy should be considered prior to active surveillance for small thyroid nodules (<10 mm) with suspicious nodules to prevent unnecessary active surveillance and patient anxiety (8–10).

Among several RSSs, the Chinese Artificial Intelligence Alliance for Thyroid and Breast Ultrasound proceeded to develop the C TIRADS (Chinese Thyroid Imaging Reporting and Data System) for Chinese clinical practice by using the Chinese thyroid US database (11). The Kwak TIRADS was issued by Kwak et al. (7). They have the similar categorization structure and shown adequate diagnostic performance in the detection of malignant nodules (12, 13) and are easily applicable for their relative simplicity. They also show particular differences: both the C and Kwak TIRADSs recommend the biopsy of nodules larger than 10 mm with suspicious US features, while the C TIRADS also suggests the biopsy of nodules smaller than 10 mm in special conditions. Meanwhile, the Kwak TIRADS was based on nodules larger than 10 mm, while the C TIRADS was not restricted on the size. However, to our knowledge, there is no study evaluating the feasibility of reducing the original threshold for FNA in the Kwak and C TIRADS.

Thus, we hypothesized that lowering the current threshold for FNA might be an effective strategy. First, we determined how the thresholds of C TIRADS and Kwak TIRADS were decreased. Next, we estimated and compared the diagnostic performance of the two TIRADSs with original thresholds and decreased thresholds in a large sample of surgical series.

## Methods

This study involving human participants was reviewed and approved by the scientific research and clinical trials ethics committee of The First Affiliated Hospital of Zhengzhou University of China and was granted a waiver of written informed consent for the use of data.

### Patients

From January 2010 to July 2017, 2,582 patients with 3,833 thyroid nodules consecutively underwent US examinations and thyroidectomy at our institution. The inclusion criteria were as follows (1): a preoperative US examination was performed; (2) the nodules underwent surgical pathology within 1 month after US examination. The exclusion criteria were as follows: (1) the US image data were incomplete, and (2) the nodules lacked determined pathological results. Finally, a total of 3,201 thyroid nodules in 2,146 patients were included in this study.

### Ultrasound examination and image analysis

All US examinations were performed with a 5–14-MHz linear probe and a real-time US system (TOSHIBA Aplio300). US examinations were performed by a senior radiologist (KC) with 33 years of experience in thyroid imaging. During the US examination, nodule images were generally obtained as at least on grayscale image in each transverse and longitudinal plane. Additional images were obtained to demonstrate the important US features of the nodules.

An overview and discussion session was held by a senior radiologist (KC) with 33 years of experience in thyroid imaging to establish consensus regarding the definitions of the US lexicons from the two TIRADSs, including size (the maximal diameter at US), composition (solid, predominately solid, predominately cystic, cystic, and spongiform), echogenicity (hyperechoic, isoechoic, hypoechoic, and very hypoechoic), shape (taller-than-wide), margins (smooth, irregular, lobulated, ill-defined, and extrathyroidal extension), and echogenic foci (punctate echogenic foci, macrocalcification, peripheral calcifications, and comet-tail artifacts).

Subsequently, US features were independently reviewed by two radiologists blinded to the biopsy results and the final pathological diagnoses (CF and YH, with 13 and 12 years, respectively, of clinical experience performing thyroid US scans and evaluating thyroid US images).

**Figure 1:**
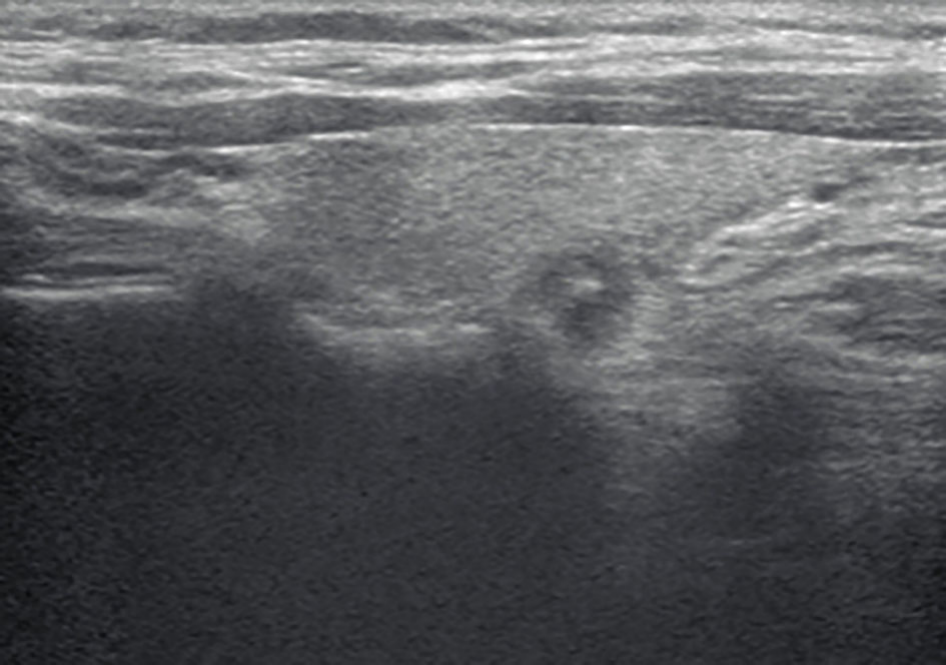
A longitudinal grayscale ultrasound (US) image obtained from a 37-year-old woman with papillary thyroid carcinoma shows a 7-mm solid, hypoechoic, and ill-defined margin thyroid nodule with punctate echogenic foci. This nodule was classified as TR4C according to the Kwak and C thyroid imaging reporting and data system (TIRADS). The original TIRADS does not recommend biopsy, while the modified TIRADS suggests biopsy.

**Figure 2:**
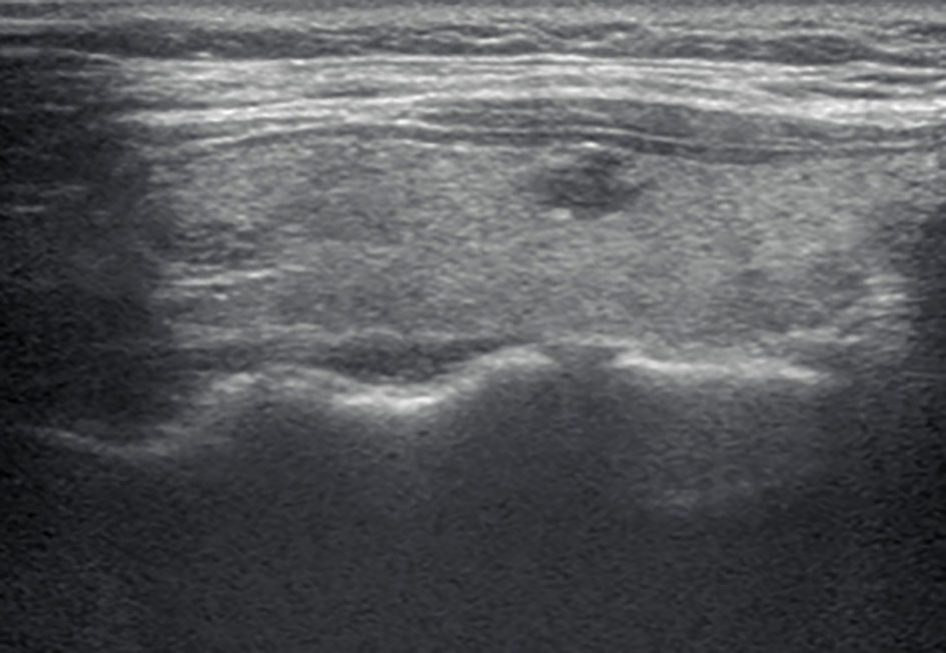
A longitudinal grayscale US image obtained from a 32-year-old woman with papillary thyroid carcinoma shows an 8 mm solid, hypoechoic, and irregular margin thyroid nodule. This nodule was classified as TR4C according to the Kwak TIRADS and TR4B according to the C TIRADS. The original TIRADS does not recommend biopsy, while the modified TIRADS suggests biopsy.

The following US features showed a significant association with malignancy in the Kwak TIRADS: solid component, marked hypoechoic/hypoechoic, irregular margins or microlobulated, microcalcifications, and taller-than-wide shape. In the C TIRADS, the said US features are as follows: solid component, marked hypoechoic, irregular margins/ill-defined or extrathyroidal extension, microcalcifications, and vertical orientation. Each suspicious US feature was assigned the same weight (one point) that is added up to a numeric score leading to a final category in the Kwak and C TIRADSs and then subtracting 1 if a negative feature of the comet tail artifacts was present in the C TIRADS (**Table 1**, **Figures 1** and **2**).

**Table 1:** The original and simulative threshold for fine-needle aspiration in the Kwak and C thyroid imaging reporting and data system (TIRADS).

	Score	Thresholds for FNA		RABM	The final simulated thresholds for FNA
		Original	Simulative		
Kwak TIRADS					
TR2	0	-	-	-	-
TR3	0	-	-	-	-
TR4A	1	≥10 mm	≥5 mm	12.1-to-1	≥10 mm
TR4B	2	≥10 mm	≥5 mm	2.5-to-1	≥10 mm
			0	2.69-to-1	
TR4C	3-4	≥10 mm	0	1-to-3.99	0
TR5	5	≥10 mm	0	1-to-9.71	0
C TIRADS					
TR2	-1	-	-	-	-
TR3	0	-	-	-	-
TR4A	1	>15 mm	>10 mm	7.4-to-1	>15 mm
TR4B	2	>10 mm	0	1-to-1.66	0
TR4C	3-4	>10 mm	0	1-to-5.97	0
TR5	5	>10 mm	0	1-to-10	0

RABM, the ratio of additional benign-to-malignant nodules being biopsied when using the simulated thresholds for FNA; The comet tail artifacts are only recorded in the absence of punctate echogenic foci of microcalcifications, and one point is subtracted to determine a final category in the C TIRADS.

### Simulative size thresholds for biopsy

The simulative thresholds for biopsy with TR4A-TR5 were confirmed using a stepwise reduction in the Kwak and C TIRADS. The ratio of additional benign-to-malignant nodules being biopsied (RABM) was calculated. The RABM equals the ratio between additional benign and malignant nodules being biopsied. If the RABM is less than 1, the decreased FNA thresholds could be accepted and are used to the modified TIRADS (hereafter modified C and Kwak TIRADSs).

### Analysis and comparison of the diagnostic performance

In a previous study, the diagnostic performances can be calculated according to indications for FNA and US-based final assessment categories. In this study, the diagnostic performances were calculated and compared between the original TIRADS and modified TIRADS according to the indications for FNA. The diagnostic performances were estimated by calculating the sensitivity, specificity, accuracy, unnecessary biopsy rate (the number of benign nodules among those recommended for biopsy), and missed malignancy rate (MMR; the number of malignant nodules among those not recommended for biopsy).

In addition, thyroid nodules were dichotomized into two groups, <10 and ≥10 mm in diameter. The diagnostic performances were calculated and compared between the two groups according to US-based final assessment categories by calculating the sensitivity, specificity, and accuracy and defining the cutoffs for ≥ TR4B as suspicious for malignancy (14).

### Statistical analysis

The demographics between benign and malignant nodules were compared by using the independent two-sample t-test for continuous data and the chi-square test for categorical data. The malignancy rates according to each category in the two TIRADSs were calculated as percentages, respectively. The diagnostic performances and unnecessary FNA rate (UFR) and MMR were calculated, along with the 95% confidence intervals, and compared by the McNemar test or Pearson test. AUCs were calculated and compared using the Z-test or DeLong test. Statistical analysis was performed with SPSS 26.0 and MedCalc 18.2.1 software. The difference was considered statistically significant at two-sided *P* < 0.05.

## Results

### Pathological diagnosis

Of 3,201 thyroid nodules, 1,727 (54.0%) were diagnosed as benign and 1,474 (46.0%) as malignant by surgical pathology. Papillary thyroid carcinomas were found to be the most common malignant nodules (1,423 papillary thyroid carcinomas, 17 follicular carcinomas, 19 medullary carcinomas, and 15 others). Adenomatous hyperplasia were the most common benign nodules (1,584 adenomatous hyperplasia, 81 follicular adenomas, 17 nodular hyperplasia, 35 inflammatory lesions, and 10 others).

### Baseline clinicopathological characteristics

The demographics and US features of the patients and nodules are summarized in **Table 2**. There were 1,411 (44.1%) nodules <10 mm and 1,790 (55.9%) nodules ≥10 mm in diameter. The malignancy rates in nodules with a diameter <10 mm was higher compared with nodules ≥10 mm (56.2% vs. 43.8%, *P* < 0.05). Patients under 55 years old had higher malignancy rates compared with ≥55 (*P* < 0.05). Benign nodules were larger than malignant nodules (20.5 ± 15.9 mm vs. 12.8 ± 12.1 mm, *P* < 0.001). In addition, there was no statistics difference in gender for the <10 mm group compared to the ≥10 mm group while patients in the ≥10 mm group were older than those in the <10 mm group.

**Table 2:** Summary of demographic and ultrasound (US) features for the patients with thyroid nodules.

Characteristics	Final pathology		Total	*P*-value
	Benign	Malignant		
No. of nodules	1,727 (54.0)	1,474 (46.0)	3201	
Age				0.000
Mean (y)	49.6 ± 12.1	44.7 ± 11.9	47.3 ± 12.2	
Range (y)	12–82	7–80	7–82	
<55	1,153 (66.8)	1,186 (80.5)	2,339 (73.1)	0.000
≥55	574 (33.2)	288 (19.5)	862 (26.9)	
Gender				0.019
Male	375 (11.7)	372 (11.6)	747 (23.3)	
Female	1,352 (42.2)	1,102 (34.4)	2,454 (76.7)	
Size				0.000
Mean (mm)	20.5 ± 15.9	12.8 ± 12.1	17.0 ± 14.8	
Range (mm)	2.0–100.0	2.0–102.0	2.0–102.0	
<10	582 (33.7)	829 (56.2)	1,411 (44.1)	0.000
≥10	1,145 (66.3)	645 (43.8)	1,790 (55.9)	

Data in parentheses are percentages.

### Risk of malignancy according to category

The detailed risk of malignancy (ROM) according to each category in Kwak and C TI-RADS is presented in **Table 3**. All of the calculated ROMs for Kwak TI-RADS were well matched within the range of the suggested ROM, with the exception of the TR2 (7.6%, 6/78) and TR3 (4.5%, 22/482). Most of the calculated ROMs for C TI-RADS were higher than its suggested ROM.

**Table 3:** Malignancy risks according to categories in the Kwak and C TIRADS.

System and categories		Pathological diagnosis		Calculated ROM (%)	Suggested ROM (%)
		Benign (n = 1727)	Malignant (n = 1474)		
Kwak TIRADS	2	72	6	7.6	0
	3	460	22	4.5	2–2.8
	4a	379	27	6.6	3.6–12.7
	4b	440	119	21.3	6.8–37.8
	4c	354	1,064	75.0	21–91.9
	5	22	236	91.4	88.7–97.9
C TIRADS	2	52	2	3.7	0
	3	567	30	5.0	<2
	4a	536	80	12.9	2–10
	4b	403	448	52.6	10–50
	4c	167	887	84.1	50–90
	5	2	27	93.1	>90

ROM, risk of malignancy.

### The ratio of additional benign-to-malignant nodules being biopsied according to simulative size thresholds

The simulative thresholds for biopsy with TR4A-TR5 were confirmed using a stepwise reduction in the Kwak and C TIRADS. The RABM according to simulated size thresholds are summarized in **Table 1**.

If the threshold for FNA for TR4C-TR5 nodules in the Kwak TIRADS were changed from ≥10 to 0 mm, the RABM would be less than 1 (1-to-3.99 and 1-to-9.71, respectively). If the threshold for FNA for TR4B-TR5 nodules in the C TIRADS were changed from >10 to 0 mm, the RABM would be less than 1 (1-to-1.66, 1-to-5.97, and 1-to-10, respectively).

The simulative thresholds with TR5 and TR4C were accepted in Kwak TIRADS and used to establish the modified Kwak TIRADS. Equally, TR4B, TR4C, and TR5 were accepted in the C TIRADS and used to establish the modified C TIRADS.

### Comparison of the diagnostic performances between original thyroid imaging reporting and data system and modified thyroid imaging reporting and data system

**Table 4** shows the diagnostic performance in Kwak and C TIRADSs by using the FNA indicator. In Kwak TIRADS, sensitivity was higher (42.6% vs. 38.7%, *P* < 0.05), while, in C TIRADS, specificity and accuracy were higher (64.0% vs. 54.9%, 52.3% vs. 49.2%, *P* < 0.05). No significant differences in the positive predictive value (PPV), negative predictive value (NPV), and UFR were found between the two TIRADSs (47.8% vs. 44.6%, 55.0% vs. 52.8%, and 52.2% vs. 55.4%, *P* > 0.05 for all).

**Table 4:** Diagnostic performances of biopsy in the Kwak and C TIRADS.

	Sensitivity	Specificity	PPV	NPV	Accuracy	UFR	MMR
Kwak TIRADS	42.6% (40.0–45.3)	54.9% (52.7–57.1)	44.6% (42.1–47.2)	52.8% (50.4–55.3)	49.2% (47.5–51.1)	55.4% (52.8–57.9)	47.1% (44.9–49.6)
C TIRADS	38.7% (36.2–41.2)	64.0% (61.6–66.2)	47.8% (44.9–50.8)	55.0% (52.9–57.2)	52.3% (50.6–54.3)	52.2% (49.2–55.1)	44.9% (42.9–47.2)
*P*-value	0.030	0.000	0.105	0.182	0.013	0.105	0.182

Numbers in parentheses are 95% confidence intervals; PPV, positive predictive value; NPV, negative predictive value; UFR, unnecessary FNA rate; MMR, missed malignancy rate.

The diagnostic performances were calculated according to FNA thresholds and were compared between the original TIRADS and the modified TIRADS (**Table 5**). The modified C TIRADS had higher sensitivity, PPV, NPV, accuracy and had a lower unnecessary biopsy rate and MMR than C TIRADS (95.1% vs. 38.7%, 61.7% vs. 47.8%, 92.3% vs. 55.0%, 70.6% vs. 52.3%, and 38.3% vs. 52.2%, 7.7% vs. 44.9%, P < 0.05 for all). However, higher specificity was found in C TIRADS (64.0% vs. 49.7%, P < 0.05). Similar trends were seen in Kwak TIRADS versus modified Kwak TIRADS.

**Table 5:** Comparison of the diagnostic performances between original TIRADS and modified TIRADS.

Systems	Sensitivity	Specificity	PPV	NPV	Accuracy	UFR	MMR
Kwak TIRADS	42.6% (40.0–45.3)	54.9% (52.7–57.1)	44.6% (42.1–47.2)	52.8% (50.4–55.3)	49.2% (47.5–51.1)	55.4% (52.8-57.9)	47.1% (44.9-49.6)
mKwak TIRADS	94.1% (92.9-95.3)	45.0% (42.6-47.5)	59.4% (57.2-61.3)	89.9% (87.7-91.8)	67.6% (66.0-69.2)	40.6% (38.7-42.6)	10.1% (8.1-12.1)
*P*-value	0.000	0.000	0.000	0.000	0.000	0.000	0.000
C TIRADS	38.7% (36.2-41.2)	64.0% (61.6-66.2)	47.8% (44.9-50.8)	55.0% (52.9-57.2)	52.3% (50.6-54.3)	52.2% (49.2-55.1)	44.9% (42.9-47.2)
mC TIRADS	95.1% (94.0-96.1)	49.7% (47.4-52.1)	61.7% (59.8-63.7)	92.3% (90.4-94.0)	70.6% (69.0-72.2)	38.3% (36.2-40.3)	7.7% (6.0-9.5)
*P*-value	0.000	0.000	0.000	0.000	0.000	0.000	0.000

Numbers in parentheses are 95% confidence intervals; PPV, positive predictive value; NPV, negative predictive value; UFR, unnecessary FNA rate; MMR, missed malignancy rate; mKwak TIRADS, modified Kwak TIRADS; mC TIRADS, modified C TIRADS.

### Comparison of diagnostic performances between the “<10 mm” and “≥10 mm” groups

We calculated the diagnostic performances in the two groups (<10 and ≥10 mm) according to US-based final assessment categories (**Table 6**).

**Table 6:** Diagnostic performance in different thyroid nodule diameters between the Kwak and the C TIRADS.

Systems	Sensitivity	Specificity	PPV	NPV	Accuracy	AUC
Kwak TIRADS						
<10 mm	97.7% (96.6-98.7)	47.3% (43.3-51.0)	72.5% (69.7-75.1)	93.5% (90.8-95.9)	76.9% (74.6-79.0)	0.837 (0.816-0.856)
≥10 mm	94.4% (92.7-96.0)	55.5% (52.7-58.4)	54.5% (51.7-57.4)	94.6% (92.9-96.3)	69.6% (67.5-71.7)	0.863 (0.843-0.876)
*P*-value	0.001	0.001	0.000	0.495	0.000	> 0.05
C TIRADS						
<10 mm	94.8% (93.2-96.4)	60.5% (56.7-64.6)	77.4% (74.8-79.8)	89.1% (85.8-92.2)	80.7% (78.5-82.8)	0.840 (0.820-0.859)
≥10 mm	89.3% (86.8-91.6)	70.1% (67.5-72.7)	62.7% (59.5-65.6)	92.1% (90.4-93.9)	77.0% (75.1-79.0)	0.860 (0.847-0.879)
*P*-value	0.000	0.000	0.000	0.084	0.013	> 0.05

Numbers in parentheses are 95% confidence intervals; PPV, positive predictive value; NPV, negative predictive value.

No significant differences in the AUC and NPV were found between the two groups in Kwak TIRADS (0.837 vs. 0.863 and 93.5% vs. 94.6%, respectively, *P* > 0.05 for all) and C TIRADS (0.840 vs. 0.860 and 89.1% vs. 92.1%, respectively, *P* > 0.05 for all). The <10 mm group had higher accuracy, sensitivity, and PPV and lower specificity than the ≥10 mm group in the two TIRADSs.

## Discussion

In our study, the simulative thresholds for biopsy with TR4A-TR5 were confirmed using a stepwise reduction in the Kwak and C TIRADSs. Then, they were used to the modified TIRADS (modified C and Kwak TIRADSs). The modified TIRADS showed higher sensitivity, PPV, NPV, and accuracy and a lower MMR and an unnecessary biopsy rate than the original TIRADS. Thus, our results suggested that the biopsy of all nodules with TR4C-TR5 in the Kwak TIRADS and TR4B-TR5 in the C TIRADS might be an effective strategy, regardless of the nodule dimension.

In some researchers, it is supported that biopsy should be considered prior to active surveillance for subcentimeter thyroid nodules with suspicious nodules to prevent unnecessary US monitoring and patient anxiety (8–10). The plausibility of biopsy first and US monitoring later equated with the plausibility of reducing the threshold for FNA. If the simulative thresholds for FNA were changed from ≥ 10 to ≥ 5 or 0 mm, the ratio of additional benign to malignant nodules being biopsied (RABM) would be less than 1. This suggests that in the setting of potential US monitoring, it would be reasonable for patients with subcentimeter nodules to undergo FNA so that those with malignant cytology could undergo US monitoring, and those with benign could be reassured.

In our study, the simulative thresholds for biopsy were confirmed using a stepwise reduction. Then, the RABM were calculated respectively. If the threshold for FNA with TR4C-TR5 nodules in the Kwak TIRADS were changed from ≥10 to 0 mm, the RABM would be less than 1. If the threshold for FNA for TR4B-TR5 nodules in the C TIRADS were changed from >10 to 0 mm, the RABM would be less than 1. In a previous study, the simulative threshold for biopsy for TR5 nodules in the TIRADS published by the American College of Radiology (ACR TI-RADS) was changed from ≥ 1.0 to ≥ 0.5 cm; the RABM was 1 (15). These simulative thresholds were used to the modified TIRADS. Upon further calculation on the diagnostic performance, the modified Kwak and C TIRADSs had superior diagnostic performance over the original TIRADS as well as a lower MMR and an unnecessary FNA rate.

The aforementioned results indicated that the biopsy of all nodules with TR4C-TR5 in the Kwak TIRADS and TR4B-TR5 in the C TIRADS might be an effective strategy. Meanwhile, it is supported that biopsy might be considered prior to active surveillance for subcentimeter thyroid nodules with TR4C-TR5 nodules in the Kwak TIRADS and TR4B-TR5 nodules. A recent study demonstrated similar findings, comparing five US-based RSSs in pediatric populations (16). Some studies also suggested that FNA should be more often performed (9, 17) which could be the reduced MMR. These changes can make the management of thyroid nodules a bit easier by using the Kwak and C TIRADSs. After the diagnosis of microcarcinoma (smaller than 10 mm) without any high-risk features (as pathological lymph nodes), we offered two management options, US monitoring or surgery, and we asked patients which option they would prefer. However, we recommend US monitoring as the first-line management because of the favorable data regarding active surveillance. On the contrary, benign nodules with a confirmed cytologic diagnosis were reassuring results for patients, which could reduce their psychological anxiety and economic burden.

The diagnostic superiority of modified TIRADS may be explained by the fact that Kwak and C TIRADSs have efficient diagnostic performances in the thyroid nodules smaller than 10 mm. Our results showed that the AUC of Kwak and C TIRADSs was similar in the <10 mm group and ≥10 mm group. Meanwhile, the diagnostic performances of the <10 mm group had higher sensitivity, accuracy, and PPV than those of the ≥10 mm group in both Kwak and C TIRADS. Simone-Schenke et al. have also demonstrated that Kwak TIRADS seemed to be a promising tool to reliably assess the risk of malignancy of subcentimeter thyroid nodules (18). Qi Qi et al. have also obtained that the AUCs of the C TIRADS and Kwak TIRADS were not statistically different between “<10 mm” and “≥10 mm” groups (12). These results suggested that Kwak and C TIRADSs were also efficient in the discrimination of malignancy in the thyroid nodules smaller than 10 mm by using US-based final assessment categories. Furthermore, in our study sample, both Kwak and C TI-RADS seemed to provide a reliable stratification of high-suspicion nodules. The calculated risk of malignancy of TR4c and TR5 was 75.0% and 91.4% in the Kwak TIRADS and 84.1% and 93.1% in the C TIRADS.

Some limitations of our study should be considered. First, the nodules included in this study were confirmed by surgical pathology, which might lead to partial bias. However, this study’s results of the diagnostic capacity of the classifications are not biased by the inherent inaccuracy of FNA cytohistology results. The FNA diagnosis includes a percentage of undetermined lesions during general populations whose final results (benign or malignant) were unknown since surgery was not performed on all of them. Second, it is a single-center experience in a tertiary referral hospital; patients coming for diagnosis and/or treatment mostly have malignant diseases. Thus, the proportion of malignant nodules in our study was higher (46.0%) than that in other studies (range, 10.3%–25.8%) (8, 19–21). Third, the interreader agreement among radiologists assessing thyroid nodules’ US features and thyroid cancer risk stratification was not evaluated in this study. Our further prospective studies will estimate interreader agreement.

In conclusion, this study suggested that the biopsy of all nodules with TR4C-TR5 in the Kwak TIRADS and TR4B-TR5 in the C TIRADS might be an effective strategy, regardless of the nodule dimension. This paper contributes to the contradiction concerning whether to perform FNA for the nodules smaller than 10 mm. Further studies should be replicated using other RSSs to estimate the rationality of biopsy of thyroid nodules smaller than 10 mm.

## Data availability statement

The original contributions presented in the study are included in the article/supplementary material. Further inquiries can be directed to the corresponding author.

## Ethics statement

The studies involving human participants were reviewed and approved by the scientific research and clinical trials ethics committee of The First Affiliated Hospital of Zhengzhou University of China. Written informed consent from the participants’ legal guardian/next of kin was not required to participate in this study in accordance with the national legislation and the institutional requirements.

## Author contributions

CF had the conception and design of this study. CF and YC provided the study materials and patients. JL reviewed, analyzed, and classified the imaging data. YW and CS performed the statistical analysis. KC provided the basic information of all cases. CF wrote the manuscript. All of the authors read and approved the final manuscript.
